# Simulation, Modeling and Experimental Research on the Thermal Effect of the Motion Error of Hydrostatic Guideways

**DOI:** 10.3390/mi12121445

**Published:** 2021-11-25

**Authors:** Pengli Lei, Zhenzhong Wang, Chenchun Shi, Yunfeng Peng, Feng Lu

**Affiliations:** Department of Mechanical and Electrical Engineering, Xiamen University, Xiamen 361005, China; kenash@mail.ustc.edu.cn (P.L.); shichenchun@nbu.edu.cn (C.S.); pengyf@xmu.edu.cn (Y.P.); 19920191151156@stu.xmu.edu.cn (F.L.)

**Keywords:** hydrostatic guideways, thermal error, finite element simulation, motion errors

## Abstract

Hydrostatic guideways are widely applied in ultra-precision machine tools, and motion errors undermine the machining accuracy. Among all the influence factors, the thermal effect distributes most to motion errors. Based on the kinematic theory and the finite element method, a 3-degrees-of-freedom quasi-static kinematics model for motion errors containing the thermal effect was established. In this model, the initial state of the closed rail as a “black box” is regarded, and a self-consistent setting method for the initial state of the guide rails is proposed. Experiments were carried out to verify the thermal motion errors simulated by the finite element method and our kinematics model. The deviation of the measured thermal vertical straightness error from the theoretical value is less than 1 μm, which ensured the effectiveness of the model we developed.

## 1. Introduction

High-precision and large-diameter aspheric optical components play a key role in advanced optical systems. The typical application areas are inertial confinement nuclear fusion devices and large telescopes. Laser nuclear fusion technology is a cutting-edge technology that uses inertial confinement to achieve controllable nuclear fusion. The National Ignition Facility (NIF) is the largest laser inertial confinement nuclear fusion device in the world today [1,2], whose optical array includes 192 laser beams. More than 7000 large-aperture optical originals are required. In recent years, research on NIF devices has also made breakthrough progress [3,4]. In this context, the research and development of high-quality and high-efficiency processing equipment for large-aperture optical components are imminent.

Ultra-precision manufacturing technology and equipment are important means to achieve mass production of those optical components. The processing order of these large-aperture optical components is usually “grind first, then polish” [5]. First, the aspheric surface directly forms via the ultra-precision grinding processing method. The optical components are then polished by the conformal polishing process. Eventually, the high-precision polishing method is utilized to further minimize the surface error. Therefore, high efficiency and high surface accuracy are realized in the grinding process, which helps to shorten the processing cycle. To this end, our research group has successfully participated in the development of UPG80, UPG60, and other series of ultra-precision grinding machines for optical components [6], all of which adopt hydrostatic guide rails.

Due to their high precision, high rigidity, and high load-bearing characteristics, the hydrostatic guideways are widely applied in the field of ultra-precision machine tools [7,8]. The friction pair interface of the hydrostatic guideway is separated by a layer of oil film, which avoids dry friction and abrasion. The thickness of the oil film generally ranges from 10 μm to 40 μm [9]. The well-known, large-scale optical ultra-precision grinding machine OAGM 2500 adopts hydrostatic guide rails for both the X-axis and Y-axis [10].

There has been abundant research in the field of motion accuracy of hydrostatic guides. Park [11,12] proposed the transfer function method to study the movement error of the worktable of the hydrostatic guideway. Zha [13] established a static analysis model of a four-pad open hydrostatic guideway, by simplifying the oil film at the oil pad into a linear spring unit. Afterwards, Zha [14] took the open hydrostatic guideway of a gantry grinder as an analysis object and proposed an effective method for compensation of motion errors. Xue [15] took the four-pad closed hydrostatic guide rail as the theoretical analysis object and studied the error averaging effect. Shi [16] analyzed the motion straightness theory of hydrostatic guideways with a quasi-static model, concluding that the motion error of the worktable is affected by surface profiles of both the upper and lower guide rails in the closed hydrostatic guideway, and the lower guide rail dominates [17].

All the studies mentioned above focus on the geometric error of the hydrostatic guide rail system. However, sufficient research has shown that the thermal deformation also contributes to the processing error of ultra-precision machining distinctly, up to 30–70% [18]. For the moving parts of the machine tools, the translational axis contains six motion errors [19]. The thermal deformation of the machine tool will cause the spatial movement of the moving parts, which directly results in the positioning error, the angular error, and the straightness error. Therefore, the study on motion accuracy considering the thermal effect is urgently required for the practical application of the hydrostatic guideway. Liu [20] studied the thermal deformation of machine tool components. Zhang [21] established a model of the relationship between spatial error and thermal factors. Liu [22] introduced the research progress of the methods of avoiding thermal errors and the methods of thermal error compensation.

The finite element method has a wide range of applications in the research of static pressure systems. Jeon [23] used the finite element method to characterize the oil film between the moving parts and the surface in the hydrostatic guide rails. Shamoto [9] also conducted static analysis on the relationship between the reaction force of the oil pads and the geometric error of the guide rails based on the finite element method.

Therefore, based on the kinematic model, combined with the thermal deformation of the hydrostatic guideway in the actual process, and supported by the finite element simulation and the experimental data of the hydrostatic platform, a 3-degrees-of-freedom (3-DOF) quasi-static kinematics model for motion errors containing the thermal effect for the hydrostatic guideway is established. Experimental results show that the model has a certain effect on thermal error prediction.

## 2. Modeling

**Figure 1:**
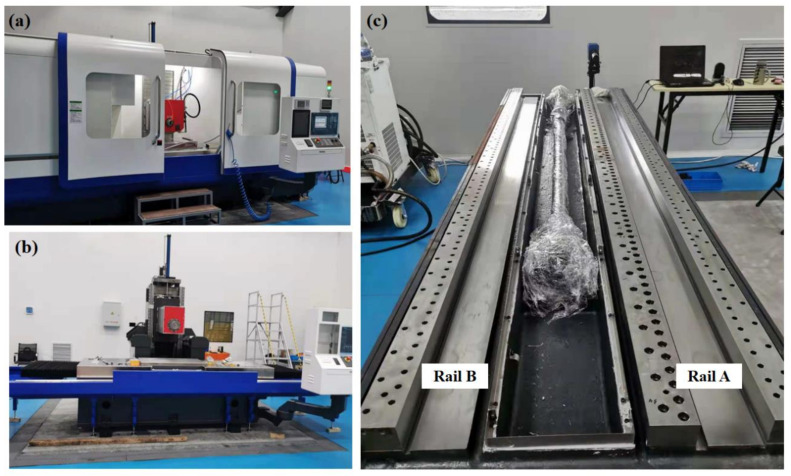
UPG60 ultra-precision grinding machine. (**a**) With the shield. (**b**) Without the shield. (**c**) Structure of the X-axis guideway.

**Figure 2:**
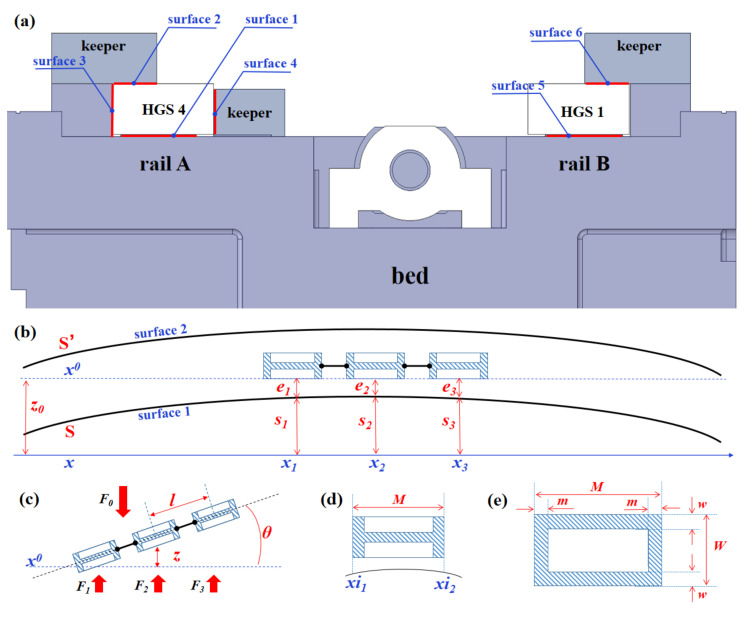
Modeling of the closed hydrostatic guideway with six pads. (**a**) View of the closed hydrostatic guideways along X-axis. (**b**,**c**) Kinematics of the three HGSs on the same side of the worktable. (**d**,**e**) detailed parameters of the HGS.

**Figure 3:**
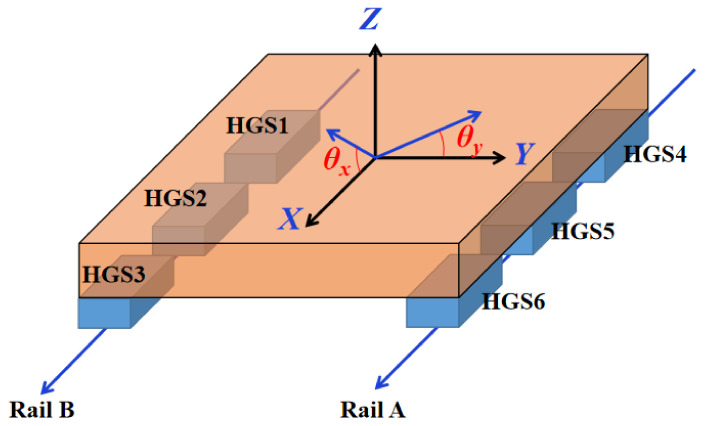
Three-degrees-of-freedom (3-DOF) kinematics model of the worktable.

The ultra-precision grinding machine UPG60 developed by our research group shown in Figure 1a,b, is the structure with the shield removed. The three linear axes of the machine are closed hydrostatic guideways with Progressive Mengen (PM) flow controllers as the restrictors. In this paper, we took the X-axis of UPG60 as the analyzed object and carried out a series of experimental studies by setting up a hydrostatic experimental platform. Figure 1c shows the X-axis structure of the grinding machine UPG60, which contains two closed hydrostatic guide rails and a worktable with six hydrostatic guide shoes (HGSs). Rail A has four rail surfaces: the bottom rail surface 1, the top rail surface 2, and the side rail surfaces 3 and 4, while Rail B has only two rail surfaces, the bottom rail surface 5 and the top rail surface 6, as shown in Figure 2a. The worktable with six HGSs is shown in Figure 3. It should be pointed out that, only HGS1 and HGS2 have four oil pads, and the other four HGSs are all guide shoes with only two oil pads. The hydrostatic guide shoes on the same side of the worktable are equally spaced, and the six HGSs are adjusted repeatedly during assembly until the plane accuracy meets the design requirements. The movement of the hydrostatic worktable can be driven by the rotation of the screw rod, and the six HGSs together with the guide rails are completely separated by a layer of oil film in the working state, so the worktable is suspended in a closed hydrostatic guide system. The motion error of the hydrostatic worktable is affected by both the parameter of the restrictors and the surface shape of the guide rails.

Figure 2 shows the principle of a single-sided closed hydrostatic guide rail, which contains three hydrostatic guide shoes. When modeling the motion errors, we mainly analyzed the thickness of the oil film of the pads. Among them, *S* is the profile curve of the bottom guide rail, while *S′* is the profile curve of the top. *x* represents the reference coordinate axis of the X-axis, and *x*_0_ is the reference axis parallel to the bottom surface of the three HGSs. Then, *z*_0_ represents the relative value of the vertical motion error of the worktable. At the same time, *e*_1_, *e*_2_, and *e*_3_, respectively, represent the average oil film thickness of the bottom oil pads of the three HGSs, which are:

(1) $$\begin{array}{c} {\bar{e}}_{i}=z\pm \frac{l_{i}}{2}\cdot \sin\theta +\int_{xi_{1}}^{xi_{2}}\frac{\left(z_{0}-S\right)}{M\cdot \cos\theta }dx\\ \approx z\pm \frac{l_{i}}{2}\cdot \theta +\int_{xi_{1}}^{xi_{2}}\frac{\left(z_{0}-S\right)}{M}dx \end{array}$$

Among them, *z_i_* is the vertical motion straightness of the i-th guide shoes, and *θ* is the deflection angular error. 

*M*-*2 m*, *W, W*-*2 w* represents the length and width of the oil pad, respectively, as shown in Figure 2e. According to References [15,24], it is believed that the topographic error of the guide rail in the width direction is relatively small, and only the topography error in the length direction of the guide rail is taken into consideration. So, *S_A_* and *S_B_* are used to define the topography error functions of Guide Rail A and Guide Rail B, respectively. 

In the quasi-static process, the worktable is in a state of equilibrium with force and moment [16]. Therefore, the overall load *F*_0_ of the worktable and the oil film reaction force *F_i_* of the oil pads satisfy the following equations:

(2) $$\begin{array}{l} \sum_{i}F_{i}=F_{0}\\ \sum_{i}F_{i}\cdot l_{i}=0 \end{array}$$

The oil film reaction force corresponding to each HGS is the resultant force of the oil film reaction forces of the top and bottom oil pads, namely:

(3) $$F_{i}=p_{ri}^{bottom}\cdot A_{i}^{bottom}-p_{ri}^{top}\cdot A_{i}^{top}$$

Among them, $p_{ri}^{bottom}$ is the reaction pressure of the bottom oil pad of the i-th guide shoe, while $p_{ri}^{top}$ is the reaction pressure of the top oil pad of the i-th guide shoe. $A_{i}^{bottom}$ is the effective bearing area of the bottom oil pad of the i-th guide shoe, and $A_{i}^{top}$ represents that of the top pad.

As introduced above, the 6 HGSs are all equipped with PM flow controllers. There is a detailed study on the characteristics of PM flow controllers in References [25,26]. According to References [16,17], the oil film pressure of the hydrostatic guide shoes with PM flow controller is:

(4) $$p_{ri}^{}=\frac{Q_{0}\cdot R_{hi}}{1-\frac{Q_{0}\cdot (K_{r}-1)\cdot R_{hi}}{P_{s}}}$$

Among them, *Q*_0_ represents the initial flow corresponding to the pressure of the oil pad is zero; *K_r_* is one of the characteristic parameters of the PM flow controller, which represents the specific flow ratio; *P_s_* is the oil supply pressure of the hydraulic station; and *R_hi_* is the flow resistance at the oil pad, whose mathematical expression is as follows:

(5) $$R_{h}=\frac{3\eta }{h^{3}\cdot (\frac{M-m}{2w}+\frac{W-w}{2m})}$$

Here, *η* refers to the dynamic viscosity of the hydrostatic oil, and *h* can be replaced by the average oil film thickness *e_i_* at the i-th oil pad. Then, we use *M*_1_, *m*_1_, *W*_1_, and *w*_1_ to represent the parameters of the top pads and *M*_2_, *m*_2_, *W*_2_, *w*_2_ to represent the parameters of the bottom pads. Thus, the oil film reaction force can be expressed as:

(6) $$\begin{array}{cc} F_{i} & =\frac{Q_{0}\cdot 3\eta }{{\bar{e_{i}}}^{3}\cdot (\frac{M_{2}-m_{2}}{2w_{2}}+\frac{W_{2}-w_{2}}{2m_{2}})-Q_{0}\cdot (K_{r}-1)\cdot \frac{3\eta }{P_{s}}}\cdot (M_{2}-m_{2})\cdot (W_{2}-w_{2})\\ & -\frac{Q_{0}\cdot 3\eta }{{\bar{e_{i}}}^{3}\cdot (\frac{M_{1}-m_{1}}{2w_{1}}+\frac{W_{1}-w_{1}}{2m_{1}})-Q_{0}\cdot (K_{r}-1)\cdot \frac{3\eta }{P_{s}}}\cdot (M_{1}-m_{1})\cdot (W_{1}-w_{1}) \end{array}$$

At the same time, it should be pointed out that when the shape of Guide Rail A and Guide Rail B are different (generally), the motion error of the worktable can be represented by the vertical motion error *z* and the two angular errors *θ_x_* and *θ_y_*, as shown in Figure 3. 

Therefore, the expression of the average oil film thickness of the bottom pads of the 6 HGSs are shown as follows:

(7) $$\begin{array}{l} {\bar{e}}_{1}=z-l\cdot \theta_{x}-l_{y}\cdot \theta_{y}+\int_{x1_{1}}^{x1_{2}}\frac{\left(z_{0}-S_{A}\right)}{M}dx\\ {\bar{e}}_{2}=z-l_{y}\cdot \theta_{y}+\int_{x2_{1}}^{x2_{2}}\frac{\left(z_{0}-S_{A}\right)}{M}dx\\ {\bar{e}}_{3}=z+l\cdot \theta_{x}-l_{y}\cdot \theta_{y}+\int_{x3_{1}}^{x3_{2}}\frac{\left(z_{0}-S_{A}\right)}{M}dx\\ {\bar{e}}_{4}=z-l\cdot \theta_{x}+l_{y}\cdot \theta_{y}+\int_{x4_{1}}^{x4_{2}}\frac{\left(z_{0}-S_{B}\right)}{M}dx\\ {\bar{e}}_{5}=z+l_{y}\cdot \theta_{y}+\int_{x5_{1}}^{x5_{2}}\frac{\left(z_{0}-S_{B}\right)}{M}dx\\ {\bar{e}}_{6}=z+l\cdot \theta_{x}+l_{y}\cdot \theta_{y}+\int_{x6_{1}}^{x6_{2}}\frac{\left(z_{0}-S_{B}\right)}{M}dx \end{array}$$

Among them, *l_y_* is the distance between the two guide rails. Combined with the oil pressure expression, by solving the force balance and moment balance equations, a set of parameters (*z*, *θx*, *θy*) can be obtained, which are the motion errors of the moving parts under the 3DOF model. The detailed parameters of the model are shown in Table 1.

## 3. Experiment

**Experimental setup:** We designed a single-axis, high-precision, hydrostatic guideway reliability and accuracy retention verification platform, through which we could monitor and measure the temperature, displacement, and motion error of the worktable during the experiment. 

The purpose of the experiment is to explore the performance of the accuracy of the static pressure rail under different temperature conditions. Collecting temperature information and motion error data at the same time in the experiment, we could then calculate the corresponding results through the theoretical model. Comparison of theoretical predictions and experimental data can further verify the accuracy of the theoretical model and its scope of applications. We therefore hope to provide theoretical support for the thermal error compensation of the hydrostatic guideway by verifying the accuracy of the model.

**Figure 4:**
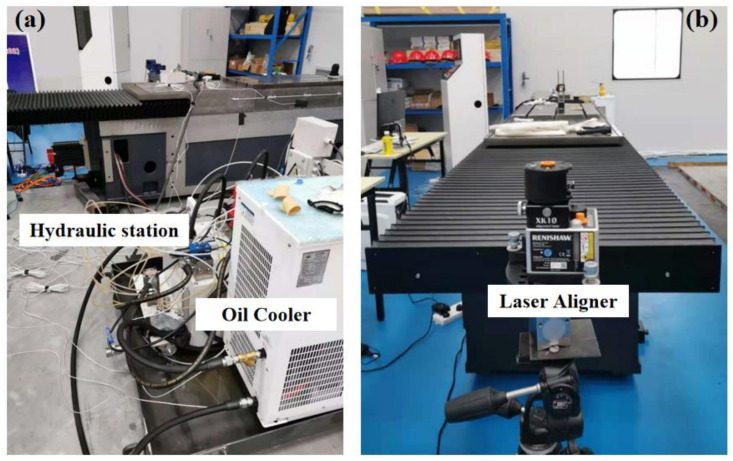
Experimental platform of the hydrostatic guideway. (**a**) Hydraulic station and the oil cooler. (**b**) Hydrostatic guideway.

The platform is shown in Figure 4. The temperature data of the key distribution points of the hydrostatic rail platform was measured by a group of temperature sensors (PT100 platinum thermal resistance; measuring range: −70 °C–500 °C; sensitivity: 0.1 °C). The motion straightness was measured by the Renishaw XK10 alignment laser system, and the angular error was measured by an electronic level meter.

The experimental platform was equipped with a hydraulic station system and a hydrostatic oil cooling circulation system. Among them, the hydraulic oil converged into the oil storage tank from the oil outlet of the guide rail and then entered the cooler for cooling, and the oil outlet of the cooler also flowed into the oil storage tank (that is, the return oil of the guide rail and the cooling oil are mixed and cooled in the oil storage tank at the same time). Finally, the oil was output to the guide rail through a hydraulic pump. All experiments were performed under isolated constant temperature conditions to avoid environmental temperature fluctuations from affecting the temperature monitoring data of the rail platform. By adjusting the cooling parameters of the oil cooler, we set up two sets of experiments.

**Experimental results:** In the data collected by the temperature sensor, we only took the temperature of the oil inlet of the guide rail, the temperature of the oil outlet of the guide rail, the temperature of the oil storage tank, and the temperature of the environment as the main analysis objects. It can be seen that the cooling parameters of the oil cooler in the two experiments are different. The cooling temperature of the oil cooler in the first experiment was controlled at about 17 °C, and in the second experiment, it was set to about 19 °C. The purpose was to control and compare the oil temperature of different oil inlets to explore the influence of temperature on the motion error of the worktable.

**Figure 5:**
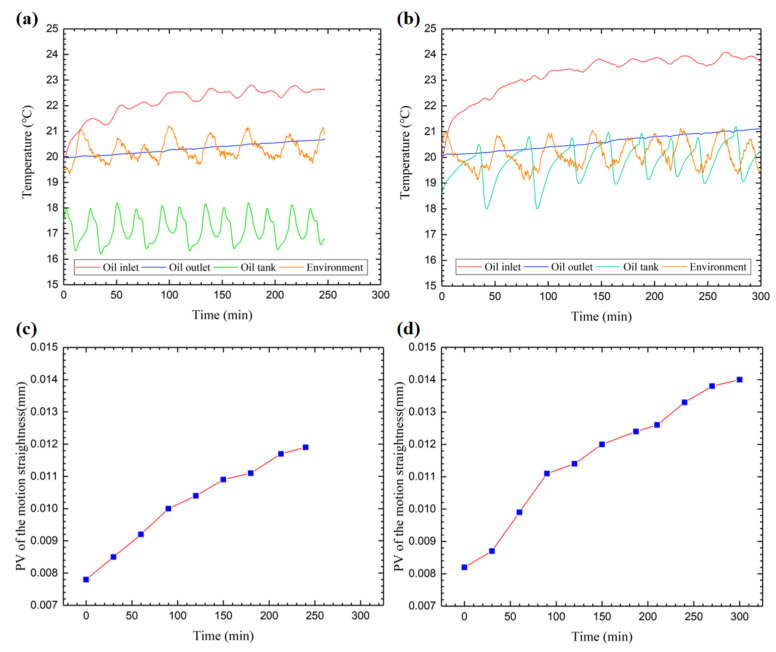
Experimental results. (**a**) Temperature monitoring data in Experiment #1. (**b**) Temperature monitoring data in Experiment #2. (**c**) Motion straightness in Experiment #1. (**d**) Motion straightness in Experiment #2.

In Figure 5a,b, the experimental results show that during the whole process, the temperature at the oil inlet of the guide rail changed most obviously, showing a trend of rising first and then stabilizing. While the temperature at the outlet continues to rise slowly, the temperature curves in the figure fluctuate, which is caused by the intermittent working mode of the oil cooler. The stable values of the temperature rising of the oil inlet in the two experiments were 2.5 °C and 3.7 °C, with a difference of about 1 °C.

Figure 5c,d reveals the trend of the peak–valley (PV) value of the motion straightness of the worktable over time. It can be seen that the vertical straightness error of the workbench gradually increases with the experimental time. At the same time, comparing the two sets of curves, it can be found that under the same initial state, the higher the oil inlet temperature, the greater the vertical straightness error of the final state. It shows that the temperature of the oil inlet is the decisive factor for the accuracy of the moving parts of the hydrostatic guideway.

**Figure 6:**
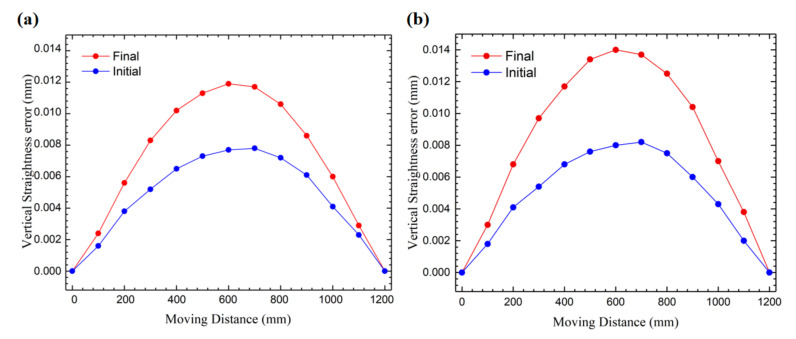
Vertical straightness error under different experimental conditions. (**a**) Experiment #1: oil temperature rises 2.5 °C. (**b**) Experiment #2: oil temperature rises 3.7 °C.

In Figure 6, we compared the initial vertical motion straightness and the final vertical motion straightness under the two experimental conditions. The measurement results show that the initial motion straightness is a convex shape (which is related to the initial topography of the rail surface of the platform), and as the experiment progresses and the temperature gradually increases, its appearance remains unchanged, but the amplitude gradually increases. Similarly, the higher the oil temperature, the higher the amplitude increase.

## 4. Discussion

To explore the characteristics of the straightness of the table motion under the influence of different oil temperatures, in this section, combined with the finite element thermal structure coupling simulation, we will use the kinematics model established in the second section to illustrate the experimental results in detail.

### 4.1. Self-Consistent Setting of the Initial State of the Guide Rails

Previous research is all based on the kinematics theory, establishing the quasi-static theoretical model of the motion error of the hydrostatic guideway. Based on the proposed model, the posture of the worktable at each position in the guide rails can be solved, and then the motion errors can be obtained. In the research process, the initial data of the surface profile of the hydrostatic guide rails were measured, and then the motion straightness of the table movement was calculated according to the established model. Reference [6] also achieved an ideal prediction result.

However, in fact, due to the hydrostatic guide rail being in a closed state during operation, and under different environmental conditions such as load or a certain operating time, the initial topography of the guide rail tends to change to a certain extent. During the normal operation of the machine tool, it is impossible to disassemble it frequently to measure the data of the guideway surface. Therefore, predicting the motion straightness based on the data measured before packaging the guide rails is still not effective. Therefore, in this paper, we propose a reverse calculation idea, that is, considering the basic accuracy of the kinematics model, combined with the measurement results of the motion straightness, a set of reasonable initial parameters of the rail surface are given. In short, we regard the closed guide rail as a “black box”, using the established kinematics model, combined with the “external” measurement results, and infer a set of reasonable “black box” initial parameters. We use that set of initial parameters to predict the motion accuracy under other conditions. We call this process from effect to cause the “self-consistent setting of the initial state of the guide rails”.

The surface profile of the guide rails is usually characterized by a chord function. For example, in the calculation in Reference [6], a set of sine functions is used to express the surface parameters of the guide rail before fine grinding. In this paper, we set the following function as the initial rail surface profile:

(8) $$S_{ini}=A_{s}+B_{1}\cdot x+B_{2}\cdot x^{2}+B_{3}\cdot x^{3}+B_{4}\cdot x^{4}$$

**Figure 7:**
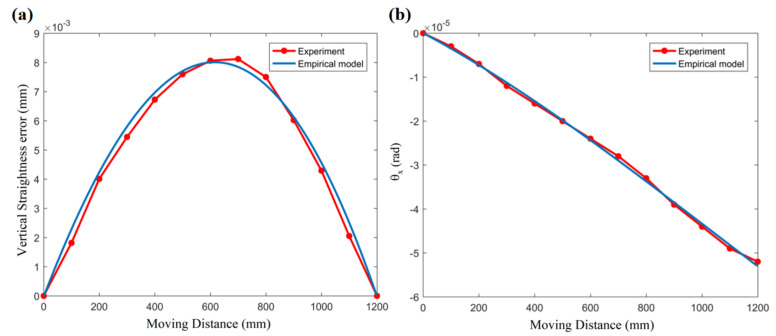
Initial motion errors. (**a**) Initial vertical straightness of the worktable. (**b**) Initial angular motion error of the worktable.

Among them, *x* is the length coordinate of the guide rail. *A_s_*, *B*_1_, *B*_2_, *B*_3_, and *B*_4_ are undetermined parameters. Further, we simplified the model conditions, considering that the parameters of Guide Rail A and Guide Rail B are the same and considering the oil film clearance to be the design value of 50 μm. According to the measurement results of the initial vertical straightness error in the third section (the blue curve in Figure 6), the theoretical results calculated by the model are close to the test results by setting a set of *A_s_*, *B*_1_, *B*_2_, *B*_3_, and *B*_4_ values. In this article, we established a 3DOF kinematics model, so in addition to the vertical motion error *z*, there are two angular errors *θ_x_* and *θ_y_* (as shown in Figure 3). Here, we take the two main error terms of *z* and *θ_x_* as the research objects. In the experiment, the angular error *θ_x_* is measured by an electronic level meter. The setting values of the initial parameters of the guide rails are shown in Table 2. Under those parameters, the comparison curve of the vertical straightness error and angle *θ_x_* calculated by the model with the measured data is shown in Figure 7.

In Figure 7, taking the parameter values in Table 2 as the input data of the “black box”, a set of vertical motion straightness error *z* and angular error *θ_x_* (set the initial value to zero) can be calculated through the kinematics model, which fit well with the experimental results. 

Here, we take the coefficient of correlation to denote the prediction accuracy of the curves in Figure 7. The mathematical expression can be given as [6]:

(9) $$R^{2}=\left(\frac{\sum \left(MD-\bar{MD})(TR-\bar{TR}\right)}{\sqrt{\sum {\left(MD-\bar{MD}\right)}^{2}{\left(TR-\bar{TR}\right)}^{2}}}\right)$$

Among them, *MD* and $\bar{MD}$ are the measured data (the red line in Figure 7) and the corresponding mean value, respectively. *TR* and $\bar{TR}$ are the predicted theoretical result (the blue line in Figure 7) and the corresponding mean value, respectively. So, the coefficient of correlation of the vertical straightness error (R_z_^2^) and the coefficient of correlation of the angular error *θ_x_* (R_a_^2^) can be calculated as 0.9785 and 0.9972, respectively. In fact, the optimal fitting parameters are more than the values given in Table 2. However, the following analysis results show that the selection of this set of parameters as the initial rail surface profile is sufficiently accurate in the prediction of thermal errors.

### 4.2. Finite Element Simulation of Thermal Deformation of Guide Rail Surface

In this section, the finite element analysis method is used to simulate the thermal deformation of the hydrostatic guideway to obtain the topographic parameters of the surface of the guideway after heating, which is used as the inputs of the model.

The material parameters are shown in Table 3. The steady-state thermal analysis module and the statics analysis module are combined to calculate the thermal deformation of the rail surface under the influence of the temperature rising of the oil film.

The meshing size is 20 mm, and the basic parts of the machine tool are set as the material of HT300, while the remaining parts are set as structural steel. The main heat sources in the working process of the hydrostatic guide rails are the power consumed during the movement of the moving guide rail and the heat generated by the linear motor. Part of the power consumed by the moving guide is the friction power consumed by the shearing oil film, and the other part is the power consumed when the hydraulic oil flows, that is, the output power of the oil pump. This consumed power will increase the temperature of the motor and the oil film and transfer heat into the moving rail through heat conduction, causing thermal deformation of the rail surface and ultimately leading to motion errors. As the motor is fixed outside of the bed, and the heat is not easy to conduct, it is not considered as the main heat source. Therefore, in the simulation process, the temperature rise of the oil film is used as the internal heat source.

When analyzing the thermal deformation caused by the internal heat source, the external heat dissipation of the machine tool must also be considered. Therefore, the load is the heating oil film of the guide rails, and the boundary condition is set as the air convection on the surface of the platform. The simulated initial temperature is 20 °C, which is a constant temperature of the environment. The convection exchange coefficient of the oil film is 300 W/(m^2^ × °C). According to the two experimental conditions, the rising of the oil film temperature during the simulation is set to 2.5 °C and 3.7 °C, respectively.

**Figure 8:**
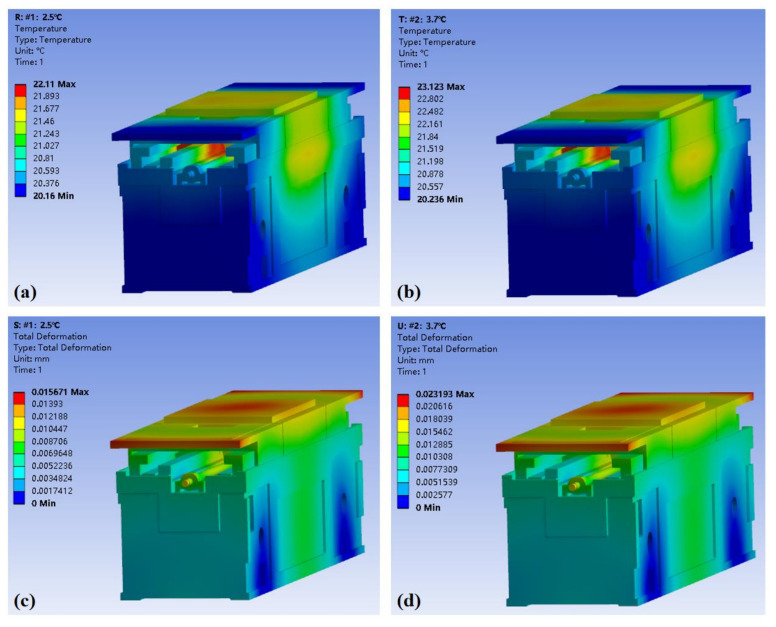
Simulation results. (**a**) Temperature field of Experiment #1, in which the temperature rise of the oil inlet is 2.5 °C. (**b**) Experiment #2, in which the temperature rise of the oil inlet is 3.7 °C. (**c**) Total thermal deformation in Experiment #1. (**d**) Total thermal deformation in Experiment #2.

It can be seen in Figure 8 that the area with the highest temperature rise is inside the guide rail, and the maximum temperature is 22.11 °C and 23.12 °C, respectively. Under the influence of this temperature field, the maximum thermal deformation of the whole experimental platform is 15.7 μm and 23.2 μm, respectively.

**Figure 9:**
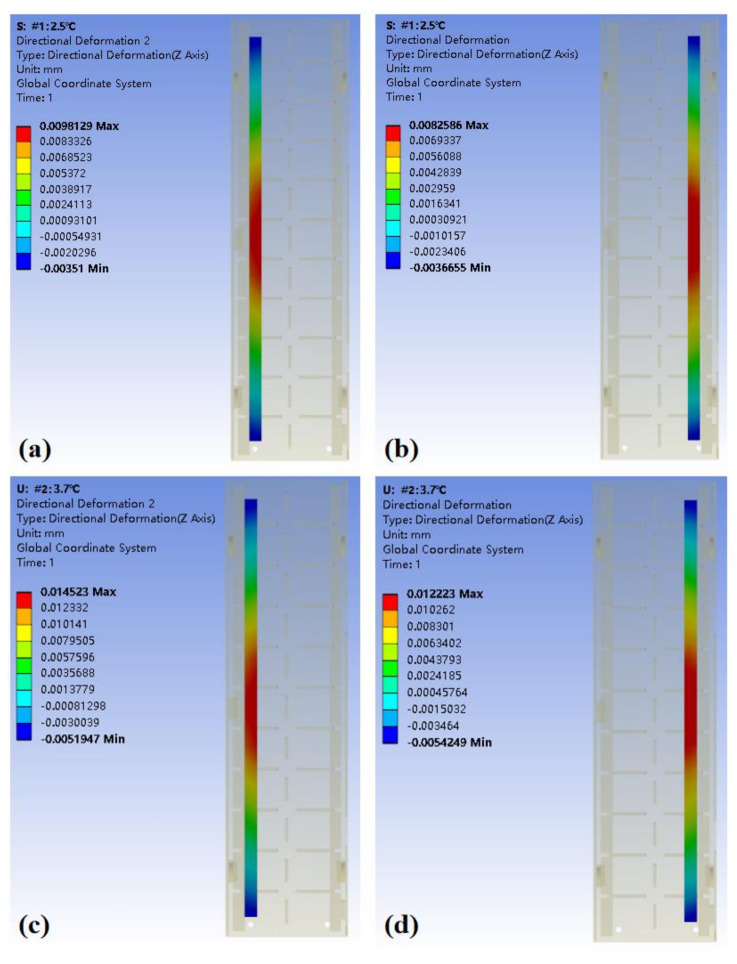
Thermal deformation of the rail surface. (**a**) Surface deformation of Rail A in Experiment #1; (**b**) Surface deformation of Rail B in Experiment #1; (**c**) Surface deformation of Rail A in Experiment #2; (**d**) Surface deformation of Rail B in Experiment #2.

In order to explore the influence of thermal deformation on the hydrostatic guide rails, we extracted the deformation data of the two guide rails, namely the deformation of the bottom surface of Guide Rail A and Guide Rail B and the deformation of the corresponding top surface, as shown in Figure 9. The simulation results show that the rail surface becomes convex after being heated. The greater the temperature rise of the oil film, the greater the deformation of the guide rail surface. 

**Figure 10:**
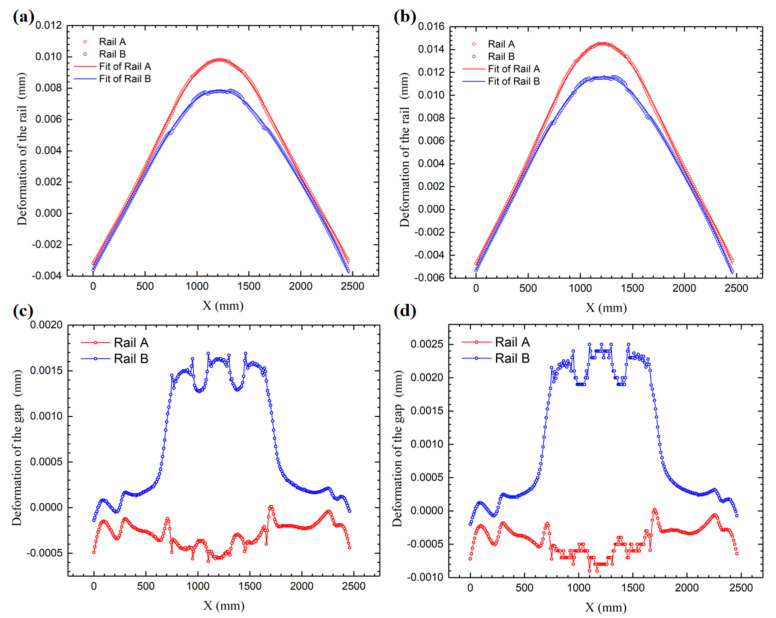
Simulation results and the fitted curve. (**a**) Fitting curve of the thermal deformation of the guide rail surface in Experiment #1; (**b**) Fitting curve of the thermal deformation of the guide rail surface in Experiment #2; (**c**) Deformation of the oil film clearance in Experiment #1; (**d**) Deformation of the oil film clearance in Experiment #2.

Further, we made the curve shown in Figure 10 through the polynomial fitting. The thermal deformation fitting curve of the rail surface in Figure 10a can be expressed as follows:

(10) $$\begin{array}{cc} \Delta S_{A}(2.5{}^{\circ}C) & =-0.00328+1.42831\times 10^{-5}\cdot x-1.86401\times 10^{-8}\cdot x^{2}+5.15349\times 10^{-11}\cdot x^{3}\\ & -5.31123\times 10^{-14}\cdot x^{4}+2.11543\times 10^{-17}\cdot x^{5}-2.93378\times 10^{-21}\cdot x^{6}\\ \Delta S_{B}(2.5{}^{\circ}C) & =-0.0036+1.2941\times 10^{-5}\cdot x-6.58874\times 10^{-9}\cdot x^{2}+2.00016\times 10^{-11}\cdot x^{3}\\ & -2.42081\times 10^{-14}\cdot x^{4}+1.02108\times 10^{-17}\cdot x^{5}-1.45877\times 10^{-21}\cdot x^{6} \end{array}$$

Similarly, the fitting curve of the thermal deformation of the guide rail surface in Figure 10b can be expressed as follows:

(11) $$\begin{array}{cc} \Delta S_{A}(3.7{}^{\circ}C) & =-0.00486+2.11799\times 10^{-5}\cdot x-2.77544\times 10^{-8}\cdot x^{2}+7.65134\times 10^{-11}\cdot x^{3}\\ & -7.87634\times 10^{-14}\cdot x^{4}+3.13557\times 10^{-17}\cdot x^{5}-4.34737\times 10^{-21}\cdot x^{6}\\ \Delta S_{B}(3.7{}^{\circ}C) & =-0.00532+1.90975\times 10^{-5}\cdot x-9.45497\times 10^{-9}\cdot x^{2}+2.90384\times 10^{-11}\cdot x^{3}\\ & -3.5359\times 10^{-14}\cdot x^{4}+1.49358\times 10^{-17}\cdot x^{5}-2.13443\times 10^{-21}\cdot x^{6} \end{array}$$

Figure 10c,d, respectively, corresponds to the difference between the thermal deformation of the top surface of the guide rail and that of the bottom surface of the guide rail, which equals the oil film gap value. It can also be inferred that the higher the oil film temperature, the greater the change in the oil film clearance. However, the overall change in the oil film clearance is relatively small, and it is concentrated in the middle of the guide rail.

### 4.3. Thermal Model of Motion Straightness

Here, we combine the initial rail surface profile parameters in Section 4.1 and the thermal deformation data of the rail surface in Section 4.2, that is, the expression of the rail surface profile can be shown as follows:

(12) $$\begin{array}{cc} \Delta S_{A}(2.5{}^{\circ}C) & =-0.01132+4.15244{\times 10}^{-5}\cdot x-5.67375{\times 10}^{-9}\cdot x^{2}\\ & -7.56398{\times 10}^{-12}\cdot x^{3}+1.13377{\times 10}^{-15}\cdot x^{4}\\ & -0.00328+1.42831\times 10^{-5}\cdot x-1.86401\times 10^{-8}\cdot x^{2}+5.15349\times 10^{-11}\cdot x^{3}\\ & -5.31123\times 10^{-14}\cdot x^{4}+2.11543\times 10^{-17}\cdot x^{5}-2.93378\times 10^{-21}\cdot x^{6}\\ \Delta S_{B}(2.5{}^{\circ}C) & =-0.01132+4.15244{\times 10}^{-5}\cdot x-5.67375{\times 10}^{-9}\cdot x^{2}\\ & -7.56398{\times 10}^{-12}\cdot x^{3}+1.13377{\times 10}^{-15}\cdot x^{4}\\ & -0.0036+1.2941\times 10^{-5}\cdot x-6.58874\times 10^{-9}\cdot x^{2}+2.00016\times 10^{-11}\cdot x^{3}\\ & -2.42081\times 10^{-14}\cdot x^{4}+1.02108\times 10^{-17}\cdot x^{5}-1.45877\times 10^{-21}\cdot x^{6} \end{array}$$

(13) $$\begin{array}{cc} \Delta S_{A}(3.7{}^{\circ}C) & =-0.01132+4.15244{\times 10}^{-5}\cdot x-5.67375{\times 10}^{-9}\cdot x^{2}\\ & -7.56398{\times 10}^{-12}\cdot x^{3}+1.13377{\times 10}^{-15}\cdot x^{4}\\ & -0.00486+2.11799\times 10^{-5}\cdot x-2.77544\times 10^{-8}\cdot x^{2}+7.65134\times 10^{-11}\cdot x^{3}\\ & -7.87634\times 10^{-14}\cdot x^{4}+3.13557\times 10^{-17}\cdot x^{5}-4.34737\times 10^{-21}\cdot x^{6}\\ \Delta S_{B}(3.7{}^{\circ}C) & =-0.01132+4.15244{\times 10}^{-5}\cdot x-5.67375{\times 10}^{-9}\cdot x^{2}\\ & -7.56398{\times 10}^{-12}\cdot x^{3}+1.13377{\times 10}^{-15}\cdot x^{4}\\ & -0.00532+1.90975\times 10^{-5}\cdot x-9.45497\times 10^{-9}\cdot x^{2}+2.90384\times 10^{-11}\cdot x^{3}\\ & -3.5359\times 10^{-14}\cdot x^{4}+1.49358\times 10^{-17}\cdot x^{5}-2.13443\times 10^{-21}\cdot x^{6} \end{array}$$

**Figure 11:**
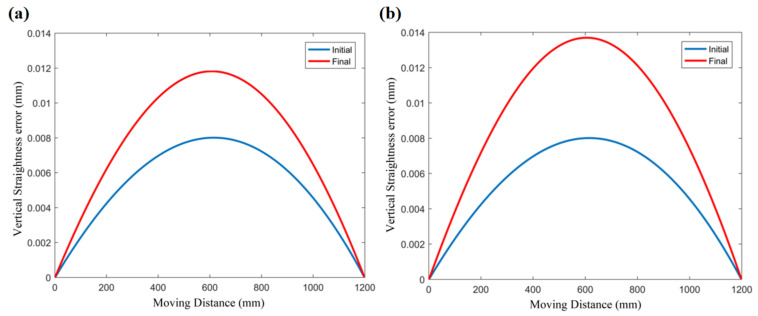
Prediction curve of the vertical straightness. (**a**) Experiment #1: temperature rise of the oil inlet is 2.5 °C. (**b**) Experiment #2: temperature rise of the oil inlet is 3.7 °C.

**Figure 12:**
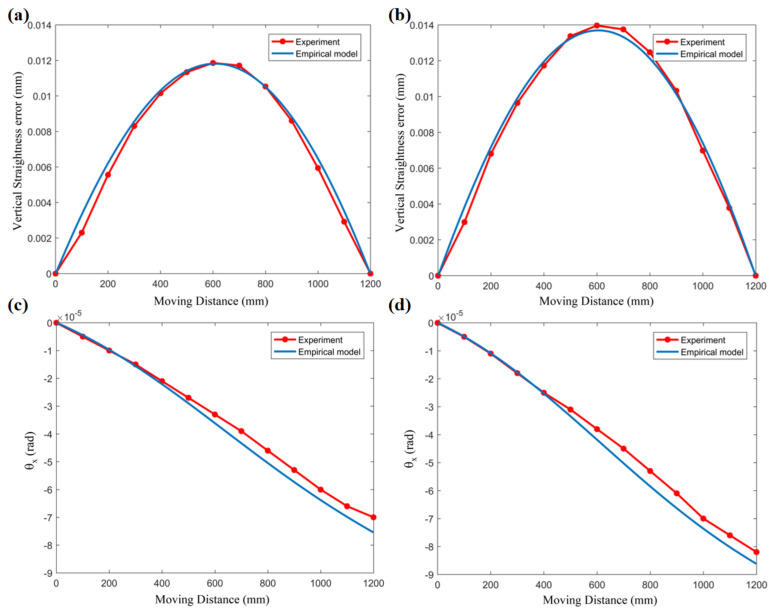
Motion errors prediction curve of comparison with the experimental data. Vertical straightness error under the condition of (**a**) Experiment #1: temperature rise of the oil inlet is 2.5 °C. (**b**) Experiment #2: oil temperature rising by 3.7 °C. Angular motion error under the condition of (**c**) Experiment #1: oil temperature rising by 2.5 °C. (**d**) Experiment #2: oil temperature rising by 3.7 °C.

Taking the above equations as the input of the model, the thermal error prediction curve of the moving parts of the hydrostatic rail can be obtained. As shown in Figure 11, Figure 11a,b represents the vertical straightness error curve of the oil film temperature rise of 2.5 °C and 3.7 °C, respectively, under the two experimental conditions. The blue curves represent the initial straightness error, and the red curves represent the vertical straightness error affected by heat. Those curves are close to the actual measured vertical straightness error data shown in Figure 12 in both trend and value.

In order to verify the accuracy of the thermal error prediction, Figure 12 shows the comparison between the theoretical results and the measured data of the motion error of the worktable. We choose vertical motion straightness *z* and angular error *θ_x_* as analysis objects. Figure 12a,c shows the comparison between the predicted results and the experimental results under the condition of the oil temperature rising by 2.5 °C. Figure 12b,d shows the comparison between the predicted results and the experimental results under the condition of the oil temperature rising by 3.7 °C. This result shows that under the same initial parameters of the guide rail, we can obtain relatively accurate predictions for the thermal influence of the motion errors under different temperature conditions.

In fact, through the following analysis, it can be found that the amplitude of the initial rail surface has little effect on the thermal influence of the motion errors. Through Equation (8) in Section 4.1, we obtained that the initial rail surface parameters satisfy:

(14) $$\begin{array}{l} S_{ini\_0}=-0.01132+4.15244{\times 10}^{-5}\cdot x-5.67375{\times 10}^{-9}\cdot x^{2}\\ -7.56398{\times 10}^{-12}\cdot x^{3}+1.13377{\times 10}^{-15}\cdot x^{4} \end{array}$$

Then, we set a series of initial rail surface shapes with different PV:

(15) $$\begin{array}{l} S_{ini\_\alpha }=\alpha \times (-0.01132+4.15244{\times 10}^{-5}\cdot x-5.67375{\times 10}^{-9}\cdot x^{2}\\ -7.56398{\times 10}^{-12}\cdot x^{3}+1.13377{\times 10}^{-15}\cdot x^{4}) \end{array}$$

**Figure 13:**
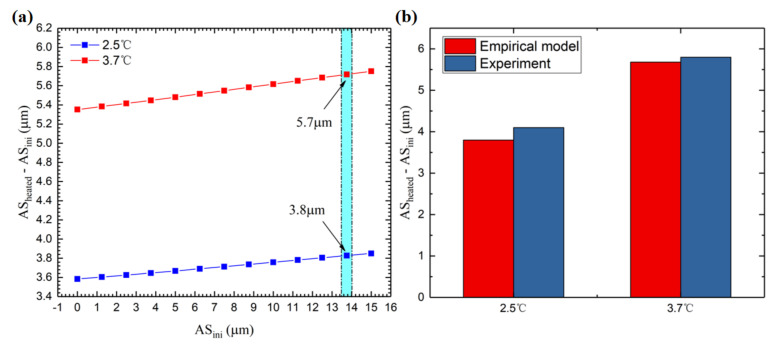
Thermal error analysis results. (**a**) Effect of the initial vertical straightness error on the thermal error. (**b**) Comparison between theoretical results and the measured data.

By changing the value of α, a series of initial rail surface shapes with different amplitudes can be obtained, and a series of corresponding initial straightness errors can be obtained through the model. Then, taking this series of initial rail surface profiles as the input, and combining it with the thermal deformation of the guide rails, we finally obtain the prediction curve of the vertical straightness error. Subtracting the amplitude of the red curve from the PV of the blue curve in Figure 11, we can obtain the difference in vertical straightness error, that is, the thermal error of motion straightness. Here, we set the α value from 0 to 1.2 so that the initial profile of the rail changes from a straight line to a curve with a PV of 34 μm. Thus, the PV range of the initial vertical straightness error is 0 to 15 μm (as shown in the horizontal axis of Figure 13). Based on those different initial PV values of the guide rail surface, combined with the thermal deformation, we can obtain a set of PV values of the initial vertical straightness error (*AS_ini_*) and the final vertical straightness error (*AS_heated_*) so that *AS_heated_* minus *AS_ini_* represents the thermal error of the vertical straightness. Then, Figure 13a can be obtained, which shows that the PV of the initial rail surface has no significant effect on the thermal error of the moving parts, and the oil temperature is the decisive factor for the thermal error. This is also the reason why the selection of a set of appropriate parameters as the initial rail surface profile is sufficiently accurate in the prediction of thermal error as mentioned in Section 4.1.

In the actual experimental measurement process, the value of the initial vertical straightness error fluctuates within a certain range, as shown in the blue area of Figure 13a. However, since the PV of the initial rail surface does not have a significant effect on the thermal error of the moving parts, we still obtained a sufficiently accurate value of the final motion error, as shown by the arrows in Figure 13a. Under the condition that the oil temperature rises 2.5 °C, the PV of the vertical straightness error of the hydrostatic worktable increased by 4.1 μm. Correspondingly, its theoretical value is 3.8 μm. Similarly, under the condition of an oil temperature rise of 3.7 °C, the PV of the vertical straightness error of the hydrostatic worktable increased by 5.8 μm, and the theoretical value is 5.7 μm. The comparison result is shown in Figure 13b.

## 5. Conclusions

In the research of this paper, firstly, a 3-DOF quasi-static kinematics model was established based on the flow characteristics of the PM flow controller and the parameters of the guide rails and guide shoes. At the same time, by setting up an experimental platform, we carried out a series of experimental studies on the thermal effect of the motion error. Comparing the measurement data of the vertical straightness error under the two experimental conditions (oil temperature rising 2.5 °C and 3.7 °C, respectively), it is shown that the initial vertical straightness error of the experimental platform is a convex shape. As the experiment progressed, the temperature of the oil increased, as did the PV value of the vertical straightness error, but the shape of the vertical straightness error remained unchanged. Similarly, the higher the oil temperature, the higher the PV increase.

On the basis of the established 3-DOF quasi-static kinematics model, we proposed a self-consistent setting method of the initial state of the guide rails so that the parameters of the initial shape of the guide rails in a closed state can be given. Furthermore, with finite element simulation, we obtained the profile of the thermal deformation of the rail surface. The motion error with the effect of heat was predicted by the 3-DOF quasi-static model, and the error between the theoretical results and the measured results on the vertical straightness error was less than 1 μm. The results show that the PV value of the initial rail surface has no significant effect on the thermal error of the moving parts, while the oil temperature is the decisive factor for the thermal error. Through the self-consistent setting method of the initial state of the guide rails, the prediction of the thermal effect of the motion error has a certain accuracy.

## Figures and Tables

**Table 1:** Parameters of the model.

Parameters	*l*				*l_y_*		Rail Length	
value	350 mm				410 mm		2460 mm	
parameters	*η*				*Q*_0_		*K_r_*	
value	0.095 × 10^−6^ N∙s/mm^2^				0.3667 × 10^−3^ mm^3^/s		2.8	
parameters	*P_s_*				*F*_0_		T	
value	3.2 MPa				9535 N		20 °C	
parameters	Top pads/mm				Bottom pads/mm			
	*M*_1_	*m*_1_	*W*_1_	*w*_1_	*M*_2_	*m*_2_	*W*_2_	*w*_2_
value	200	12	43	12	200	12	75	12

**Table 2:** Parameter setting values of the initial state of the guide rail.

Parameter	*A_s_*	*B*_1_	*B*_2_	*B*_3_	*B*_5_
value	−0.01132	4.15244 × 10^−5^	−5.67375 × 10^−9^	−7.56398 × 10^−12^	1.13377 × 10^−15^

**Table 3:** Material parameters of HT300.

Parameters	Volume Density (g/cm^3^)	Young’s Modulus (GPa)	Poisson’s Ratio	Thermal Expansion Coefficient (K^−1^)	Thermal Conductivity (Wm^−1^ K^−1^)	Specific Heat Capacity (Jkg^−1^ K^−1^)
value	6.6–7.4	115–160	0.23–0.27	8.5 × 10^−6^	39.2	470

## Data Availability

Not applicable.
